# Local spatial variations analysis of smear-positive tuberculosis in Xinjiang using Geographically Weighted Regression model

**DOI:** 10.1186/s12889-016-3723-4

**Published:** 2016-10-06

**Authors:** Wang Wei, Jin Yuan-Yuan, Yan Ci, Alayi Ahan, Cao Ming-Qin

**Affiliations:** Present Address: Department Epidemiology and Health Statistics, School of Public Health, Xinjiang Medical University, Urumqi, Xinjiang China

**Keywords:** Tuberculosis, Ordinary least square regression model, Geographically weighted regression model, Local spatial variations

## Abstract

**Background:**

The spatial interplay between socioeconomic factors and tuberculosis (TB) cases contributes to the understanding of regional tuberculosis burdens. Historically, local Poisson Geographically Weighted Regression (GWR) has allowed for the identification of the geographic disparities of TB cases and their relevant socioeconomic determinants, thereby forecasting local regression coefficients for the relations between the incidence of TB and its socioeconomic determinants. Therefore, the aims of this study were to: (1) identify the socioeconomic determinants of geographic disparities of smear positive TB in Xinjiang, China (2) confirm if the incidence of smear positive TB and its associated socioeconomic determinants demonstrate spatial variability (3) compare the performance of two main models: one is Ordinary Least Square Regression (OLS), and the other local GWR model.

**Methods:**

Reported smear-positive TB cases in Xinjiang were extracted from the TB surveillance system database during 2004–2010. The average number of smear-positive TB cases notified in Xinjiang was collected from 98 districts/counties. The population density (POPden), proportion of minorities (PROmin), number of infectious disease network reporting agencies (NUMagen), proportion of agricultural population (PROagr), and per capita annual gross domestic product (per capita GDP) were gathered from the Xinjiang Statistical Yearbook covering a period from 2004 to 2010. The OLS model and GWR model were then utilized to investigate socioeconomic determinants of smear-positive TB cases. Geoda 1.6.7, and GWR 4.0 software were used for data analysis.

**Results:**

Our findings indicate that the relations between the average number of smear-positive TB cases notified in Xinjiang and their socioeconomic determinants (POPden, PROmin, NUMagen, PROagr, and per capita GDP) were significantly spatially non-stationary. This means that in some areas more smear-positive TB cases could be related to higher socioeconomic determinant regression coefficients, but in some areas more smear-positive TB cases were found to do with lower socioeconomic determinant regression coefficients. We also found out that the GWR model could be better exploited to geographically differentiate the relationships between the average number of smear-positive TB cases and their socioeconomic determinants, which could interpret the dataset better (adjusted *R* ^2^ = 0.912, AICc = 1107.22) than the OLS model (adjusted *R* ^2^ = 0.768, AICc = 1196.74).

**Conclusions:**

POPden, PROmin, NUMagen, PROagr, and per capita GDP are socioeconomic determinants of smear-positive TB cases. Comprehending the spatial heterogeneity of POPden, PROmin, NUMagen, PROagr, per capita GDP, and smear-positive TB cases could provide valuable information for TB precaution and control strategies.

## Background

Tuberculosis (TB) now ranks alongside HIV as one dominant cause of death worldwide [1]. Without treatment, the mortality of TB is high, the same is the mortality of sputum smear-positive TB cases, and smear-positive TB cases are highly infectious [1, 2]. Studies from the pre-chemotherapy era indicated that about 70 % of people with sputum smear-positive pulmonary TB died within 10 years [1], and national tuberculosis programs concentrated on the diagnosis and treatment of sputum smear positive TB cases. In 2013, it was estimated that there were a total of 9.0 million new TB cases and 1.5 million deaths due to TB [2]. The 22 TB high-burden countries accounted for 80 % of the world’s TB cases, and China ranked second on that list, accounting for 12 % of global incidence of TB [2, 3]. Xinjiang has been confirmed as one of the high TB burdened (high TB case notification rates) provinces of China, as reported by the Chinese Center for Disease Control and Prevention (CDC) based on data collected during the period from 2010 to 2013 [4]. Although the notification rates of TB in Xinjiang have shown an absolute downward trend from 172.73/100,000 in 2010 to 164.46/100,000 in 2013, the current rate remains significantly higher than the national average [4-6].

The correlation between the average number of smear-positive TB cases and relative socioeconomic determinants has been well confirmed over the past five years. Some epidemiologic studies have pointed out that the magnitude of the problem varies across settings, possibly due to unfavorable socioeconomic conditions, overcrowding, poverty, socio-cultural barriers and HIV infection [7, 8]. Other spatial studies in China have demonstrated that population density, and economic level are latent risk factors for the spread of TB in China [9]. Xinjiang is a multi-ethnic area, with a minority population of 49.49 million out of a total 72.70 million people. Minorities in Xinjiang refer to Uygurs, Kazaks, Hui, and other 46 minority nationalities, whose total population is less than Han. The lifestyle between most of the minorities and Han differs from each other. Under such a backdrop, a large proportion of the minority population was a special determinant factor for TB prevalence in Xinjiang, compared with other provinces in China. Wubuli A successfully found a correlation between TB incidence and the proportion of minority populations in Xinjiang, but failed in explaining the geographic disparities of sputum smear-positive TB incidences and relative socioeconomic factors [5].

Historically, researches to understand the relationship between smear-positive TB and relative socioeconomic determinants have meant a great deal to public health personnel and policy makers. In China, the correlation between socioeconomic factors and TB incidences has been shown to vary geographically, especially in Xinjiang province [5, 10]. Local modeling approaches have enabled investigators to much accurately estimate the geographical differences in relations between TB incidences and socioeconomic factors. Local Geographically Weighted Regression (GWR) modeling techniques were utilized to calculate local regression coefficients, which allowed health professors to better assess how the effects of socioeconomic determinants change by geographic location [11, 12]. Thus the objectives of this study were to: (1) identify the socioeconomic determinants of the geographic disparities of smear positive tuberculosis in Xinjiang, China (2) confirm if the average number of smear-positive TB cases and relative risk factors demonstrate spatial variability in Xinjiang, and (3) compare the performances of the two models: the OLS model, and the GWR model.

## Methods

### Data sources

**Fig. 1:**
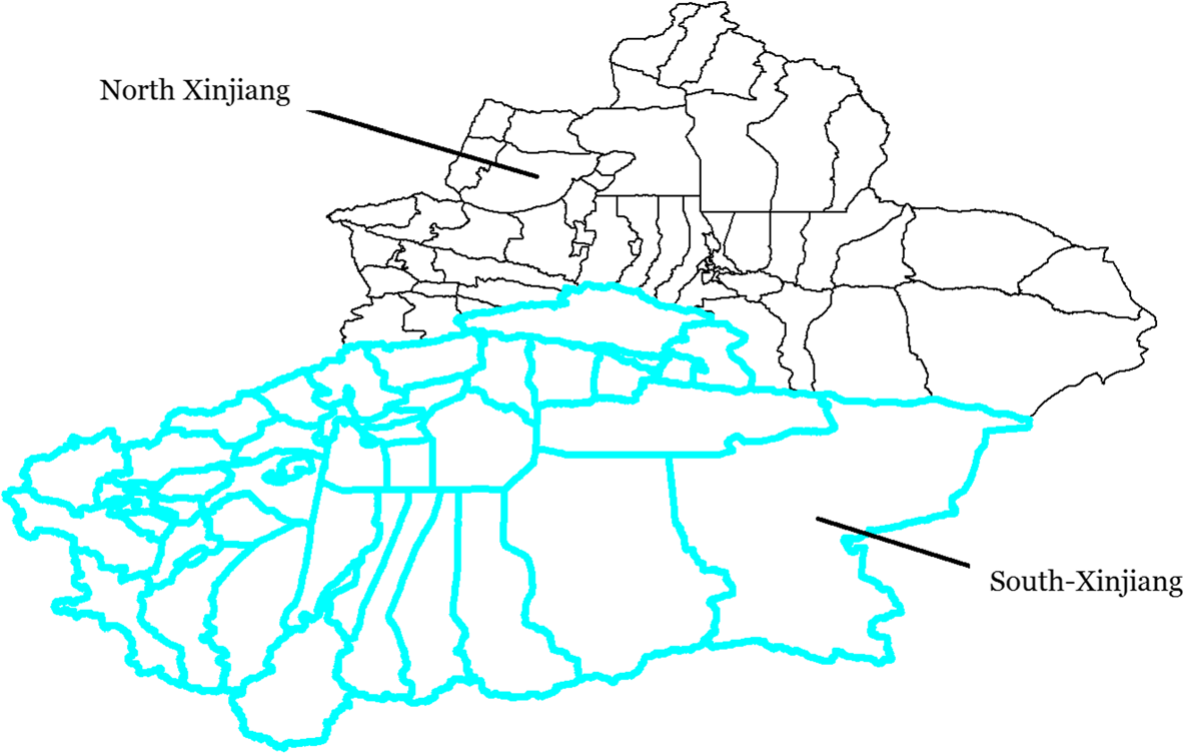
The boundary between Northern Xinjiang and Southern Xinjiang in China

A retrospective research was carried out in the province of Xinjiang which consists of 14 prefectures, and is further divided into 98 districts/counties, with an area of 1.66 million km^2^ and an estimated population of 23.22 million in 2015. Overall, Xinjiang includes two parts, Northern Xinjiang (including 43 districts/counties) and Southern Xinjiang (including 55 districts/county) (Fig. 1). A cartographic boundary file was collected from the Data Sharing Infrastructure of the Earth System Science - Xinjiang & Central Asia Science Data Center. The smear-positive TB data were gathered from the internet-based National Infectious Diseases Repotting System (NIDRS). Socioeconomic variables such as “population density (POPden, people/km^2^)”, “proportion of minorities (PROmin, percentile, %)”, “number of infectious disease network reporting agencies (NUMagen, per 1,000 people)”, “proportion of agricultural population (PROagr, percentile, %), and “per capita annual gross domestic product (per capita GDP, 10,000 RMB yuan)” were collected from the Xinjiang Statistical Yearbook, at the district/county level, for a period from 2004 to 2010.

#### Statistical analysis

This study applies the average number of smear-positive TB cases notified in Xinjiang as the measurement of TB prevalence and dependent variable, and takes the five socioeconomic determinants as independent variables. The average number of smear-positive TB cases is calculated from arithmetic mean for the total number of smear-positive TB cases during 2004–2010. Similarly, other five values of the same period are also obtained by this method, including “POPden (population at year-end/land area)”, “PROmin (population of minorities/population at year-end)”, “NUMagen (infectious disease network reporting agencies numbers/population at year-end × 1000)”, “PROagr (agricultural population /population at year-end)”, “per capita GDP”.

Firstly, the paper explained the global relations between dependent variable and independent variables in using of OLS model. The diagnoses of OLS model were achieved by the assessment of multi-collinearity and residuals (Table 1), based on the analysis of variance inflator factor (VIF). If VIF were greater than 10, this indicated multicollinearity would be verified [13]. In addition, there are other options to conduct the diagnostics of OLS model, such as Koenker (BP) statistic, Joint F-Statistic, Joint Wald Statistic, and Jarque-Bera (JB) statistic. The application of BP statistic aims to determine whether the explanatory variables in the model have a consistent relationship with the dependent variable both in geographic space and in data space. Correspondingly, the Joint F-Statistic and Joint Wald Statistic are used to measure the overall OLS model statistical significance. It is worth noting that the Joint F-Statistic is trustworthy only when the BP statistic is not statistically significant (*P* > 0.05). Otherwise, it should apply Joint Wald Statistic to determine overall model significance. JB statistic indicates whether the residuals are normally distributed or not. If p-value for BP statistic is smaller than 0.05, it would be better to carry out GWR analysis in the statistics of significant non-stationarity. Thus, when JB statistic is not statistically significant (*P* > 0.05), the OLS model will become useless and it suggests to use other model for presenting the nonlinear relationships, such as GWR model [14].

**Table 1:** OLS results

Coefficients of OLS results						
Variable	*Coefficient*	*95 % Confidence Interval*	*Probability*	*Robust t statistic*	*Robust Probability*	*VIF value*
Intercept	−144.516	(−219.025, −70.067)	<0.001	−3.533	0.001	-
NUMagen	85.618	(−92.803, 264.034)	0.349	0.937	0.351	1.415
PROagr	54.867	(−54.985, 164.719)	0.330	0.767	0.444	1.886
Per capita GDP	−23.246	(−40.265, −6.227)	<0.001	−4.167	<0.001	1.211
PROmin	200.603	(127.491, 273.715)	<0.001	3.062	0.003	1.961
POPden	0.068	(0.058,0.078)	<0.001	6.468	<0.001	1.344
Diagnostics of OLS results						
Number of Observations	98	Akaike’s Information Criterion (AICc)				1196.76
Multiple R squared	0.779	Adjusted R-Squared				0.768
Joint F statistic	67.878	Prob(>F), (5,96) degrees of freedom				<0.001
Joint Wald statistic	181.455	Prob(>chi-squared), (5) degrees of freedom				<0.001
Koenker(BP) statistic	26.564	Prob(>chi-squared), (5) degrees of freedom				<0.001
Jarque-Beta statistic	108.815	Prob(>chi-squared), (2) degrees of freedom				<0.001

The spatial independency of residuals in OLS model was evaluated by the spatial autocorrelation coefficient, namely Moran’s I, which ranges from −1 (negative autocorrelation) to +1 (positive spatial autocorrelation). Positive spatial autocorrelation (0 < Moran’s I ≤ 1) meant there were similar values in adjacent areas, while negative autocorrelation (−1 ≤ Moran’s I < 0) implied the dissimilar values at the nearby locations. Nevertheless, if there was no spatial autocorrelation (Moran’s I = 0) found, the spatial arrangement would be completely random [15]. If it found the existence of spatial autocorrelation in the residuals (Moran’s I ≠ 0), OLS model would not fit the dataset.

Considering the spatial autocorrelation in the residuals, it is fitful to use GWR model for the measurement of the smear-positive TB data. A conventional GWR model can be described as below:

$$ {y}_i={\displaystyle {\sum}_k{\beta}_k}\left({u}_i,{v}_i\right){x}_{ki}+{\varepsilon}_i $$

where *y*_*i*_ is dependent variable at location *i*, *x*_*i*_ represents *k*th independent variable at location *i*, and *ε*_*i*_ is the Gaussian error at location *i*, (*u*_*i*_, *v*_*i*_) is the x-y coordinate of the *i*th location; and coefficients *β*_*k*_(*u*_*i*_, *v*_*i*_) are varying conditions at location *i*.

The paper examined the spatial variability of an estimated local regression coefficient in order to determine whether there was spatial heterogeneity in this process [16, 13]. Additionally, through corrected Akaike Information Criterion (AICc) and adjusted coefficient of determination (Adjusted R^2^), it evaluated the comparison of OLS model and GWR model.

The analysis of this article was completed on the basis of smear-positive TB data. OLS model was processed by GeoDa 1.6.7 software with 0.05 significant levels, while GWR model was implemented by GWR 4.0, in which different Kernel Type and estimated Bandwidth Methods were attempted. It turned out the model with the lowest AIC statistic is one with the best model fit [17], according to the AIC goodness of fit statistic for comparing models. This criterion was also used to compare different GWR models and OLS model. The GWR regression coefficients of five determinants in 98 districts/counties were compared by absolute values.

## Results

**Fig. 2:**
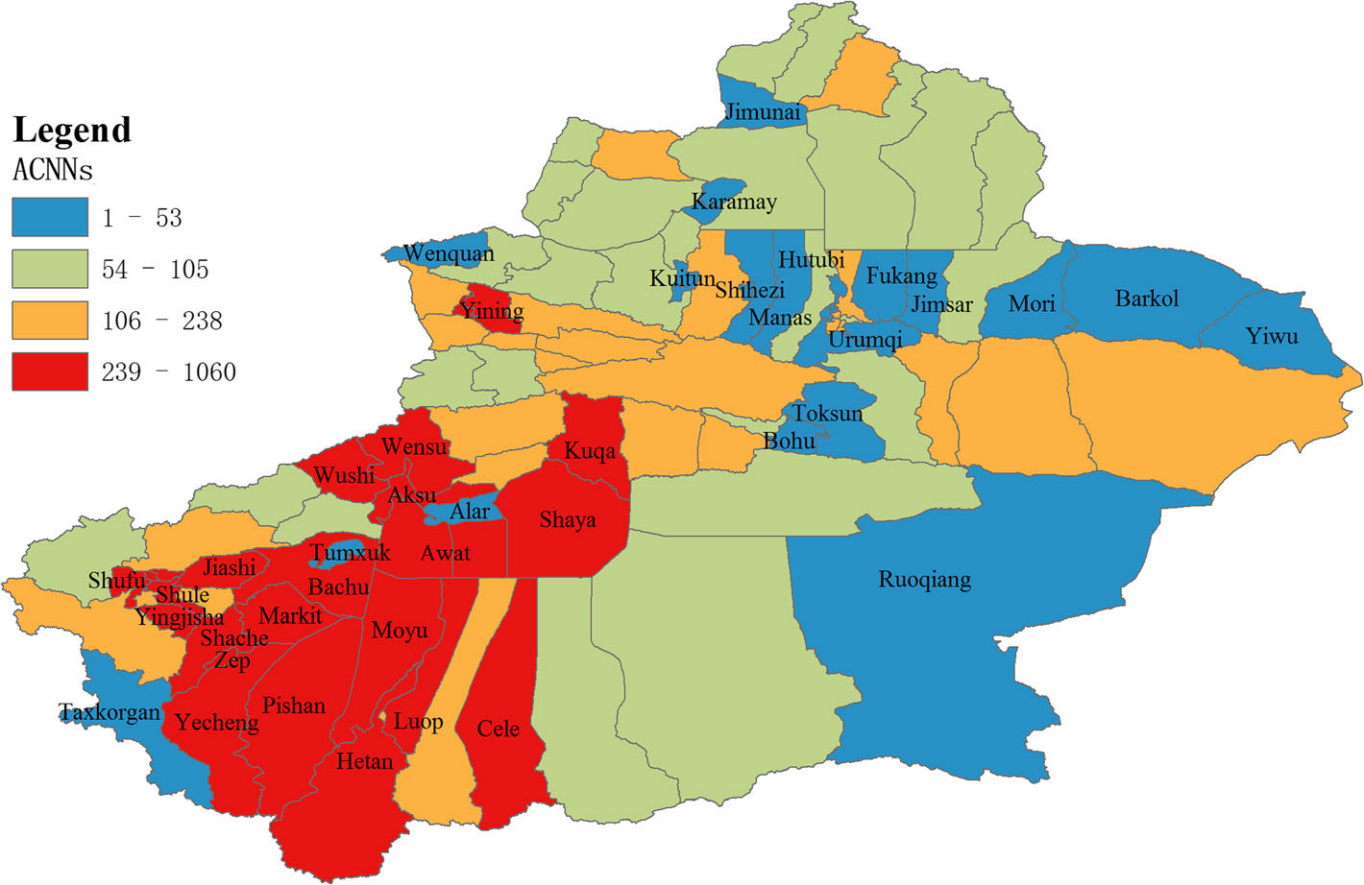
The spatial distribution of the average number of smear-positive TB cases notified in Xinjiang (ACNNs) during 2004–2010. The higher values of the average number of smear-positive TB cases were in Shule, Shufu, Yecheng, Pishan, Hetan, Lup, Cele, Shaya, KuqaWensu, et al. [Red areas]. The lower values of the average number of smear-positive TB cases were in Ruoqiang, Taxkogan, Tokxon, Bohu, Yiwu, Barkol, Wenquan, Jimunai, et al. [Blue areas]

**Fig. 3:**
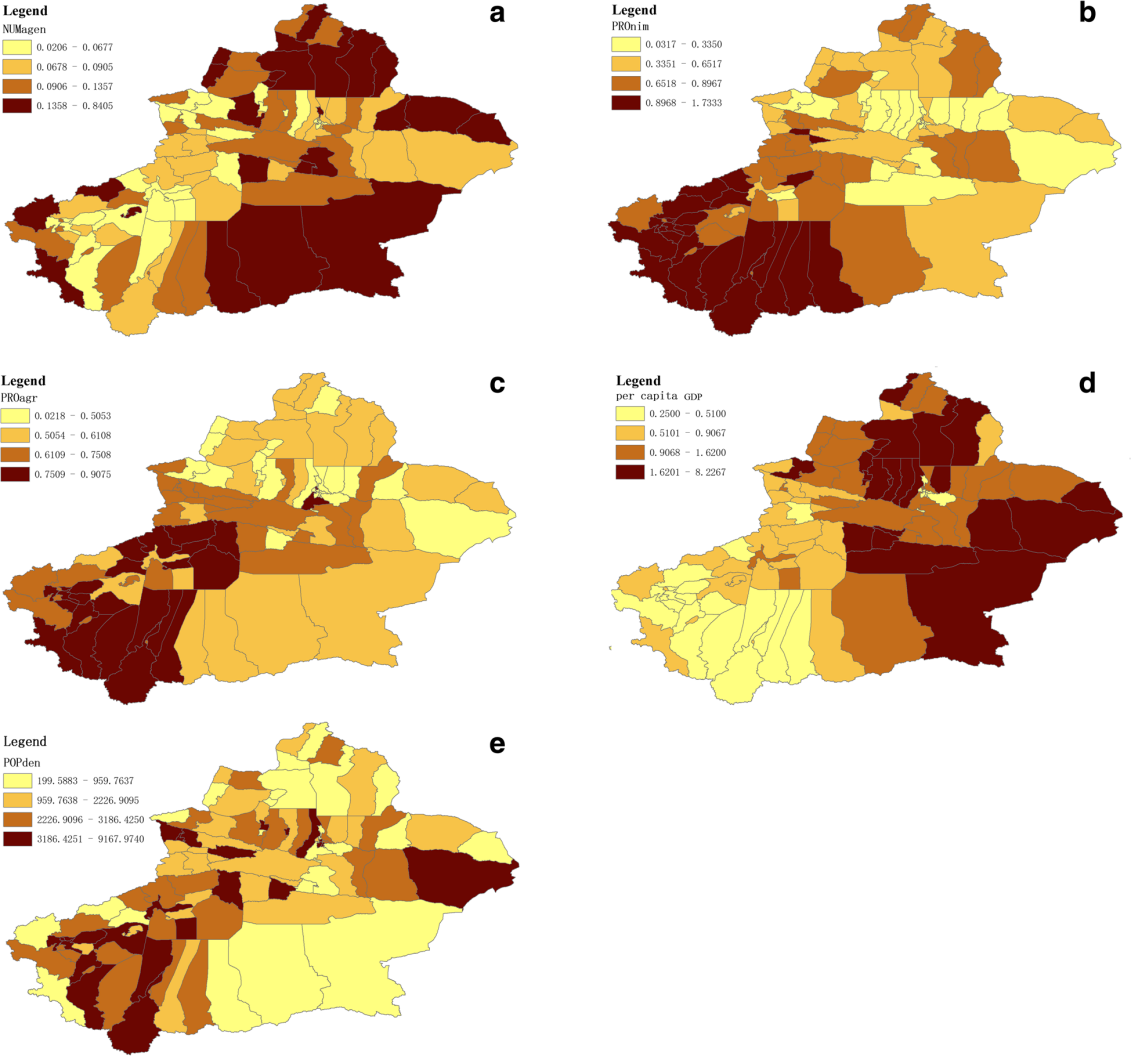
The spatial distribution of five socioeconomic determinants for the average number of smear-positive TB cases. **a** The spatial distribution of the number of infectious disease network reporting agencies (NUMagen, per 1,000 people); **b** The spatial distribution of the proportion of the minorities (PROmin, percentile, %); **c** The spatial distribution of the proportion of agricultural population (PROagr, percentile, %); **d** The spatial distribution of the per capita annual gross domestic product (per capita GDP, 10,000 RMB yuan); **e** The spatial distribution of the population density (POPden, people/km^2^)

From 2004 to 2010, a total of 105,186 new cases of smear-positive TB were reported to happen in Xinjiang, with the average number of smear-positive TB cases ranging from 1 to 1,060 (Fig. 2) during the period from 2004 to 2010, 74,065 which were in South-Xinjiang, and 31,121 in North-Xinjiang. There are huge differences in the average number of smear-positive TB cases happening among 98 districts/counties in Xinjiang. Figure 2 shows some districts/ counties located in South-Xinjiang, with more smear-positive TB cases, whereas less smear-positive TB cases occurred in some districts/ counties in North-Xinjiang. Figure 3a-e indicate the distribution of five socioeconomic determinants, for the average number of smear-positive TB cases were not identical. NUNagen (Fig. 3a) showed high values clustered in some area surrounding the border. Generally speaking, districts/counties in South-West Xinjiang had higher PROmin and PROagr values than other areas (Fig. 3b-c). The per capita GDP values in some districts/counties in North and East areas would be higher than that in other areas (Fig. 3d). Furthermore, high POPden values were found in prefecture centers (Fig. 3e).

The VIF for OLS model indicated that OLS estimations were not biased from multicollinearnity. Overall OLS model is trustworthy since Joint Wald Statistic is of significance in statistics (*P* < 0.05) (Table 1). However, the relations between the average number of smear-positive TB cases and socioeconomic determinants estimated using OLS model were weak and biased (*R* ^2^ = 0.779, adjusted *R* ^2^ = 0.7680), with p-value for BP statistic less than 0.05. After examining the residuals of OLS model, it was found that the residuals had positive spatial autocorrelation (Moran’s I = 0.0492, *P* = 0.0226). The Moran’s I indicated that there was a spatial autocorrelation of the residuals between the average number of smear-positive TB cases and related socioeconomic determinants; meanwhile, local relations couldn’t be estimated by the OLS model. We further employed GWR model to fit the data, since the existence of dependent residuals are violated to the assumptions of OLS model [13].

Five items, namely POPden, PROmin, NUMagen, PROagr, per capita GDP were analyzed as predictors for the average number of smear-positive TB cases by means of different GWR models. GWR model with Adaptive Kernel and AICc Bandwidth was selected, due to its lowest AIC statistic and higher R^2^ (Table 2). The summary of GWR model is listed in Table 3. As indicated by the results, GWR model is more suitable than OLS model, because GWR model has much higher adjusted R^2^ values (*R* ^2^ = 0.9284, adjusted *R* ^2^ = 0.9123) and much lower AIC coefficients (AICc = 1107.2245) than OLS model (AICc = 1196.7364), implying that the GWR model is a significant improvement on the OLS model for all continents. And GWR model could explain 92.84 % of total model variation.

**Table 2:** Combinations between different Kernel type and Bandwidth method

Gaussian kernel type	Neighbors/Bandwidth	Bandwidth method	Residual squares	Sigma	AICc	*R* ^2^	Adjusted *R* ^2^
Fixed	5.623	AICc	264203.073	55.644	1124.024	0.909	0.892
Fixed	6.217	CV	289875.259	57.707	1130.152	0.900	0.884
Adaptive	67	AICc	208499.043	50.260	1107.333	0.928	0.912
Adaptive	70	CV	216186.153	50.891	1108.955	0.926	0.910

**Table 3:** Summary of GWR results

Parameter	Min	P_25_	P_50_	P_75_	Max
Intercept	−252.083	−198.475	−49.814	−16.277	−3.855
NUMagen	−31.369	−16.635	6.156	35.418	191.178
PROagr	−75.699	−15.126	22.020	66.609	122.253
Per capita GDP	−57.321	−37.183	−13.432	−6.688	−5.882
PROmin	93.448	122.124	137.576	185.184	232.462
POPden	0.025	0.028	0.041	0.094	0.100

**Fig. 4:**
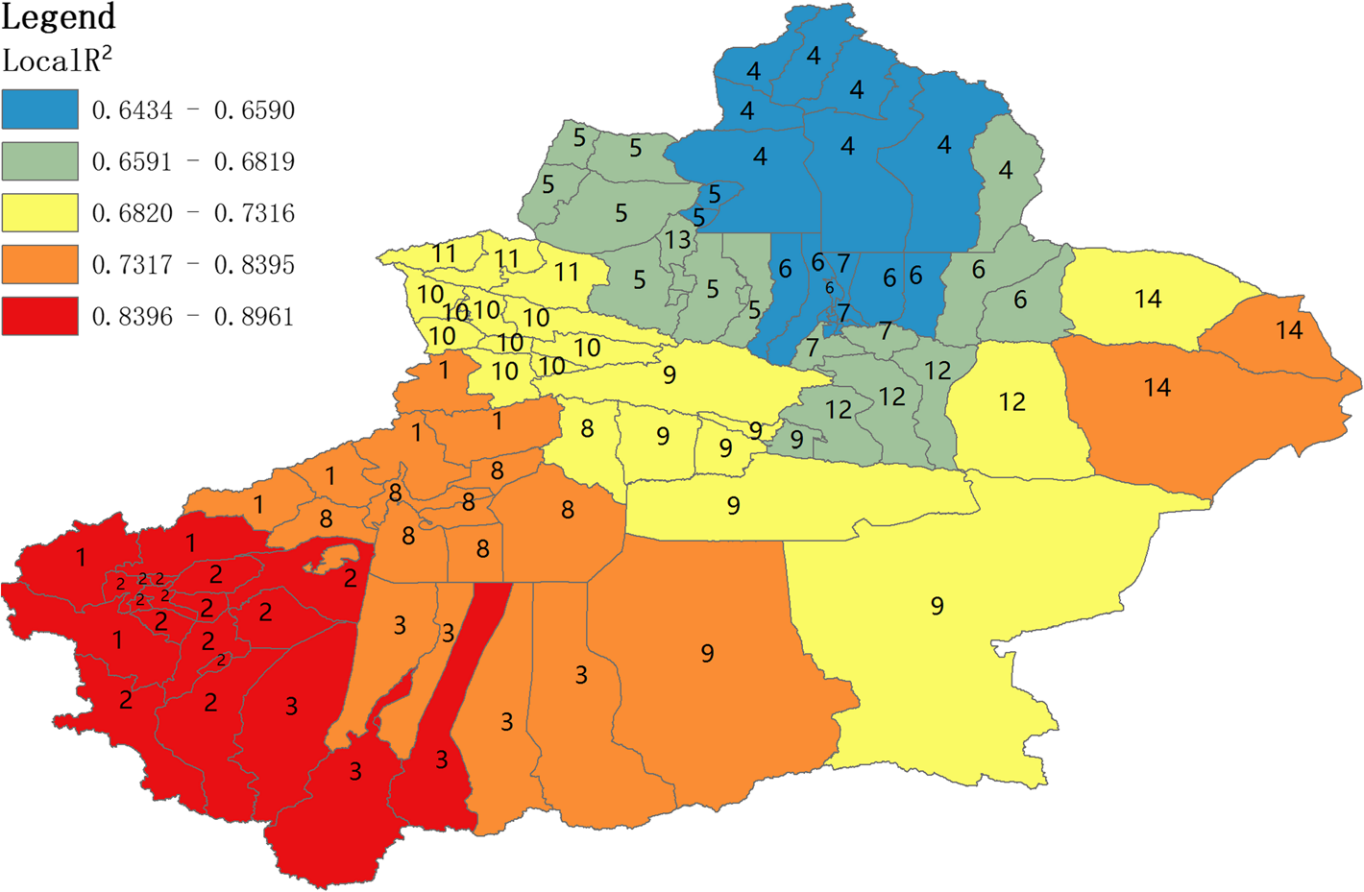
Local coefficients of determination (R^2^). Spatial mapping of local coefficients of R^2^, which were calculated based on GWR model. The maximum R^2^ values were in Kashi (prefecture 2), such as Tashkurgan Tajik. The minimum R^2^ values were in Altay (prefecture 4), such as Burqin. Number 1–14 (Kirgiz, Kashi, Hetan, Altay, Tacheng, Changji, Urumqi, Aksu, Bayingolin, Yili, Bortala, Turpan, Karamay, Hami) represented 14 prefectures in Xinjiang

Figure 4 indicated that the values of R^2^ were heterogeneously distributed in 98 districts/counties. Kirgiz (prefecture 1) is composed of 3 districts/counties, Kashi (prefecture 2) 13 districts/counties, and Hotan (prefecture 3) 3 districts/counties, which were fitted best for the overall GWR model. This model was not fitted better in Altay (prefecture 4) consisting of 7 districts/counties, Tacheng (prefecture 5) consisting of 2 districts/counties, Changji (prefecture 6) consisting of 5 districts/counties, and Urumqi (prefecture 7) consisting of 8 districts/counties.

**Fig. 5:**
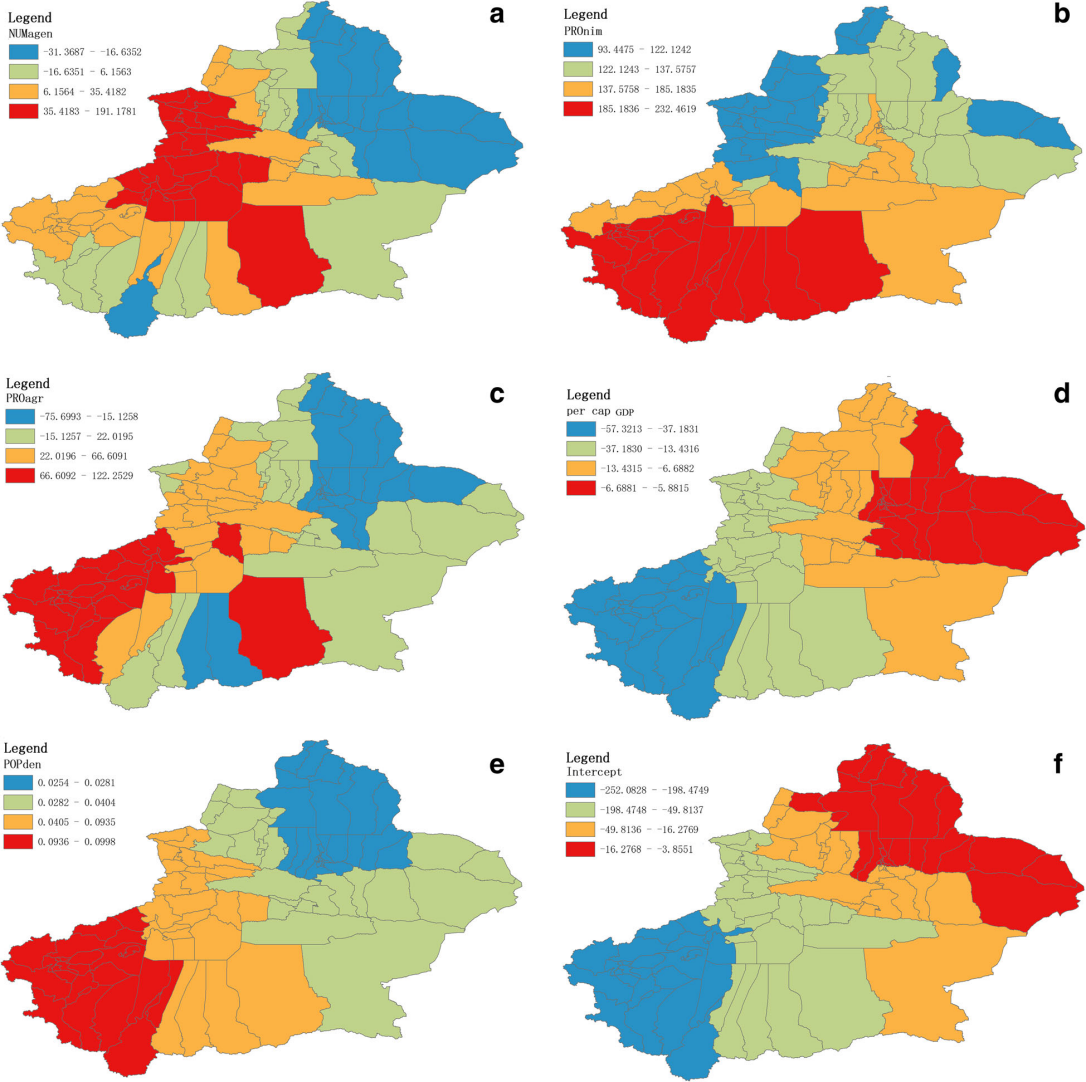
The spatial mapping of local regression coefficients (NUMagen (**a**), PROmin (**b**), PROagr (**c**), per capita GDP (**d**), POPden (**e**), and intercept (**f**)) for each district/county, based on the calculation of the Geographically Weighted Regression (GWR) model. Dependent variable was the average number of smear-positive TB cases notified in Xinjiang during the period from 2004 to 2010

The spatial variation in parameter estimation for the intercept, POPden, PROmin, NUMagen, PROagr, and per capita GDP values are shown in Fig. 5a-f. As revealed by the NUMagen coefficients distribution (Fig. 5a), Yili and Aksu [Blue areas] presented higher NUMagen values than other areas. And lower NUMagen coefficients were widely distributed in Altay, Urumqi, Hami (prefecture 14), Changji, Turpan (prefecture 12)) [Blue areas].

As indicated by the PROmin coefficients distribution (Fig. 5b), the PROmin coefficients were positive. Higher PROmin coefficients were distributed in Kashi, Hetan, and Bayingolin [Red areas], and lower PROmin coefficients in Tacheng, Yili, and partial Altay [Blue areas]. The regression coefficients of POPagr were negative in Altay, Urumqi, Changji, Turpan, and partial Hami [Blue areas], when POPagr coefficients were mostly positive in other prefectures (Fig. 5c).

The per capita GDP regression coefficients (Fig. 5d) were negative in most areas of Xinjiang. Higher per capita GDP coefficients were distributed in Hetan, Kashi, Kirgiz, and partial Bayingolin [Blue areas], and lower per capita GDP coefficients in East-Xinjiang [Red areas].

Figure 5e showed the evidence of non-stationary of POPden coefficients. The contour map of local coefficients of POPden showed that POPden coefficients were varied spatially. South-West Xinjiang (Kirgiz, Kashi) [Red areas] presented higher POPden coefficients, and North-Xinjiang (Altay, Tacheng, Changji, Urumqi) [Blue areas] lower POPden coefficients.

Figure 5f represented the spatial distribution of intercept term, indicating POPden, PROmin, NUMagen, PROagr, and per capita GDP didn’t cause impact on the average number of smear-positive TB cases. Higher intercept values lied in South-Xinjiang (Hetan, Kashi, Aksu, Kirgiz) [Blue areas]. The distribution implied that besides five socioeconomic determinants, there were still other factors bound up with the average number of smear-positive TB cases (Fig. 5f).

## Discussion

The geographical heterogeneity was detected by GWR model in terms of the relations between the average number of smear-positive TB cases and the corresponding socioeconomic determinants, since the existence of dependent residuals is violated to the assumptions of OLS model. The values of R^2^, adjusted R^2^, and AIC statistic all indicated that GWR model was fitted better for the relations. However, we also provided an evidence for the limitation of GWR model. GWR model was very sensitive to the chosen Kernel Type and Bandwidth Method. The similar finding was reported by Lin CH, with regard to the relations of entomology and dengue cases [13].

Our study also provided further indications that there were non-stationary relations between the average number of smear-positive TB cases and related socioeconomic factors in Xinjiang. It is evident that intensity and direction of NUMagen’s influence were different in Xinjiang. The NUMagen coefficients in Altay, Hami, Changji, Urumqi, Turpan were negative, whereas the coefficients were mostly positive in other areas. In South-Xinjiang, higher PROmin, PROagr, and POPden were shown to contribute to more smear-positive TB cases. The results also demonstrated that areas with higher per capita GDP were in relation to lower average number of smear-positive TB cases. Therefore, the results presented in this study indicate that the spatial interplay between five socioeconomic determinants are of vital significance to the distribution of the average number of smear-positive TB cases in Xinjiang, since a certain distribution trend of five determinants coefficients has shown the corresponding influences on the average number of smear-positive TB cases. And TB prevention and control strategy may intensify in these areas with other complexity factors, which cannot be explained by known determinants.

There are several limitations that deserve to be discussed. First, our spatial regression analysis was focused on socioeconomic determinants of the average number of smear-positive TB cases; however, other determinants of the average number of smear-positive TB cases were also essential, including geographical environment [10], climate conditions [18–20], cultural customs [21]. In terms of longitude, there were a lack of unified conclusions upon the influence of longitude. A previous finding in China showed that higher TB incidence in some areas was interrelated with higher longitude [9]. However, Vargas MH demonstrated that higher TB incidence in some areas was closely related with lower longitude [22]. And we noticed that, in Xinjiang, some areas with higher longitude (Kunlun Mountains) were related with more smear-positive TB cases, and lower longitude (Junggar Basin) more smear-positive TB cases as well. Therefore, more determinants of the average number of smear-positive TB cases should be taken into account in further studies, such as longitude [23], and average temperature [9]. Second, we collected smear-positive TB data during the period from 2004 to 2010, and the timeliness and representation of smear positive TB data may be terrible. However, we were purely concerned about the relations between socioeconomic determinants and the average number of smear-positive TB cases, and made a comparison of the fit of OLS and GWR model. Thus, the timeliness would have less impact on the relations. Thirdly, we used the average number of smear-positive TB cases as the dependent variable to estimate the regression coefficients, which would affect the fit of GWR model. Given this, further studies should utilize more TB incidence variables to estimate the regression coefficients in GWR model.

## Conclusions

In this study, the spatial heterogeneity of smear-positive TB was analyzed, and GWR model was set up, based on smear-positive TB data during the period from 2004 to 2010, as well as affecting factors. Our findings suggested that GWR model could explain the spatial variation of the dataset, whereas OLS model could not. At the same time, the chosen determinants explained the mostly variation of the average number of smear-positive TB cases during the period from 2004 to 2010. The average number of smear-positive TB cases distributed in South-Xinjiang was higher, and complexity factors affected the TB prevalence in these areas. Therefore, regional strategies aimed at TB prevention and control should be identified in accordance with the relations between socioeconomic determinants and TB.
